# The ethics of psychopharmacological research in legal minors

**DOI:** 10.1186/1753-2000-2-39

**Published:** 2008-12-08

**Authors:** Jacinta OA Tan, Michael Koelch

**Affiliations:** 1 The Ethox Centre, University of Oxford, Badenoch Building, Old Road Campus, Headington, Oxford, OX3 7LF, UK; 2 Department of Child and Adolescent Psychiatry/Psychotherapy at the University Hospital Ulm, Ulm, Germany

## Abstract

Research in psychopharmacology for children and adolescents is fraught with ethical problems and tensions. This has practical consequences as it leads to a paucity of the research that is essential to support the treatment of this vulnerable group. In this article, we will discuss some of the ethical issues which are relevant to such research, and explore their implications for both research and standard care. We suggest that finding a way forward requires a willingness to acknowledge and discuss the inherent conflicts between the ethical principles involved. Furthermore, in order to facilitate more, ethically sound psychopharmacology research in children and adolescents, we suggest more ethical analysis, empirical ethics research and ethics input built into psychopharmacological research design.

## Introduction

*"It is the duty of the physician in medical research to protect the life, health, privacy, and dignity of the human subject." *[1]

Ethics is an important issue in psychopharmacological research, especially for research amongst vulnerable patient populations. Research involving legal minors is often difficult because of the complexities of consent from legal minors and the emphasis on the protection of children and adolescents. The research into pharmacological treatments for children and young people with mental disorders is particularly fraught, because of the ethical issues surrounding research in what is in effect a doubly vulnerable group of individuals. Unfortunately, these difficulties often become barriers to conducting research with this group and pharmaceutical companies may prefer not to fund such research, giving rise to the current paucity of good research evidence. However, the lack of a good evidence base upon which to treat this vulnerable group is itself ethically reprehensible.

In this article, we will look at the background of ethics of research, the premise of research and the central ethical dilemmas and contradictions which affect paediatric psychopharmacology research. We will then touch on each of several issues which complicate the ethical dilemmas for psychopharmacology research in children and young people in practice: the premise of research, consent and competence, dilemmas of inequalities of healthcare provision, the impact of research design and the requirement for 'minimal risk' and 'benefit', and influences of commercial interests. We will suggest that the way forward is to face squarely the reasons for the restrictions placed on psychopharmacological research in minors and the inherent ethical tensions and contradictions, to consider all the ethical issues. This, together with a greater integration of ethical analysis and research into psychopharmacological research methodology, can provide a way forwards that enables good, ethically sound research to take place.

## The relevance and legacy of history

Medical research has a dark history of abuse by physicians of large numbers of vulnerable prisoners and ethnic minorities in the name of (often scientifically highly dubious) medical research. As a response to these past abuses, and the recognition of the power that those in the medical profession in particular have over patients, ethical codes and principles governing medical research have been developed as early as 1964 when the Declaration of Helsinki was made [1].

Since then, strict rules for medical research with human subjects have been developed from these ethical principles in most jurisdictions. Medical researchers are legally obliged to abide by these rules. These rules are particularly strict for researchers conducting research amongst patient populations which are vulnerable, for example prisoners, legal minors, the distressed, the mentally disordered, and those who lack competence to give consent. The historical legacy of abusive experiments with vulnerable populations, which is the current high ethical standards and legal requirements on researchers who want to conduct studies with vulnerable populations, has a cruel twist. A paradoxical situation now exists: these populations are now so well protected against research by rigorous regulatory requirements for clinical trials that the standard of routine care is much less well-founded than for the general population as there is less research data available about safety and efficacy of medication and other treatments.

In routine care, pharmacological interventions in children and adolescents continue to increase. However, the paucity of available medications specifically licensed for use in this age group, because of the lack of research evidence to support licensing, means that many drugs are used 'off-label'. 'Off-label' use means that they are used outside the bounds of the licence granted to the drug. A drug licence specifies the age range, medical indications and dosages for the use of the drug, based on data on efficacy and safety demonstrated by research. 'Off-label' use, in contrast, tends to rely on anecdote, personal experience of and confidence in using the drug in question, and consensus amongst colleagues. Benefit-risk evaluations of this 'off-label' use are therefore largely missing, with side effects and long-term aspects especially yet unknown [2-4].

'Off-label' use has now been recognized as a particular problem for drug safety [5,6]. The rate of 'off-label' use is especially high in pre-school children and less so in adolescents, but rates are generally high in comparison with adults [7,8]. In some countries, 'off-label' use poses a problem with regard to funding of treatment as health insurers generally refuse to reimburse the costs of using these drugs outside the indications of the licence [9]. Another legal and ethical aspect of 'off-label' use is who carries the responsibility for liability in the case of side effects or other adverse events [10].

It is an ethical conundrum that the need to protect vulnerable populations against research has the consequence of generating a lower standard of safety in routine care for these very patients, who arguably deserve more protection and less exposure to risk in the course of their medical treatment. Research and clinical studies are therefore urgently required in order to reduce the high rate of 'off-label' medication use in minors and improve safety [11]. These aspects of safety, dosage and the long- and short-term side effects of drugs in children and adolescents – as well as the use of these drugs during pregnancy – are frequently discussed, and the consensus is that the state of research at this time is insufficient [12]. At present the state of knowledge on both the safety and efficacy of psychotropic drugs in children and adolescents and the quality of the ethical standards varies according to the age of the patients. Again, there is a paradox that the younger the children, the more limited our knowledge and the more uncertain the benefits and risks of 'off-label' use. This is because the younger the age group, the greater the vulnerability and therefore the greater the difficulties of conducting research, and the greater the differences between a child's physiology and an adult's and therefore the less the likelihood that adult research results may be informative. Therefore the younger and more vulnerable the child, the less able clinicians are to uphold the ethical standards behind the paradigms of benefiting the patient and doing no harm [13].

As these problems have become increasingly recognized over the last decade, the feasibility and efficacy of using legislation to increase the availability of approved and safe drugs for children has been discussed in the USA and Europe. In the USA, special legal regulations have been in existence since the 1990s to encourage the development of paediatric medications by granting further years of patent protection to pharmaceutical companies in return for studies in the paediatric population [14]. This appears to have had the intended effect. Indeed, neuropharmacological medications comprise 11% of all patent protection granted for paediatric studies. Unfortunately, these regulations have not been without problems [15]. Several studies on paediatric depression were hurriedly conducted in the USA under the pressure of time before the legislation ran out. As a result, these studies are methodically flawed [16]. In Europe, similar legislation has been in effect since 2007, but the impact of the 'EU Regulation on medicinal products for use in children' has yet to be seen [17].

## The underlying premises of medical research

The historical context in which psychopharmacological research in children and adolescents takes place has been important in shaping the dilemmas of the current situation. However, the best way to consider the ethical issues in order to find a way forward is not merely to sketch out the problems as they currently exist, but to conduct an analysis of the ethical dilemmas present. The first place to begin the ethical analysis of psychopharmacology research in minors is to lay out the underlying premises of both medical research and the ethical principles that govern its oversight. We would suggest that, when simplified, the ethical reasoning for most interventional research is generally as follows and is represented in most codices concerning research such as the Declaration of Helsinki by the WMA, the National Commission for the Protection of Human Subjects of Biomedical and Behavioral Research which published in 1976 their report about research on prisoners, and in 1979 the Belmont Report [1,18]. Most other codices or regulations rely on these 'basic' codices.

1. The medical profession has a duty to treatment each patient in his or her best interests, by offering the best treatment available and knowing what is the best as well as the safest treatment for the patient's condition – this is Duty of Care, [1] (see Appendix 1),

2. Research is therefore required to provide the knowledge base in order to fulfil our Duty of Care to our patients [1] (see Appendix 2);

3. Where possible we should do research without using human subjects but in many cases we do have to use human subjects;

4. If we do research in patients, we should only involve vulnerable patients if there is no alternative group that can be used and the research is necessary [19] (see Appendix 3);

5. For a vulnerable patient group, protection in their best interests is the rule. Even when such patients give consent or assent, research should only be permitted if it causes minimal harm or distress and has the potential to benefit each patient in the future or others with the same condition. Along the same lines of protection in best interests, only therapeutic research that has a clear prospect of benefiting the individual participant to at least the same extent of the current alternative treatments is permitted to carry more than a minimal risk of harm or distress to subjects [19-21] (see Appendix 4).

6. But: research always has its own risks of harm and research is only justified if there is uncertainty in the outcome, harm or benefit which needs to be determined, which means that may be as much likelihood of harm or lack of benefit from participation as there is likelihood of benefit;

7. Furthermore, research designs and research physicians are inherently unable to prioritise the interests of the individual participant to the same extent as in normal medical care because their primary objective is to answer a research question and they must follow research protocols;

8. Therefore taking part in research can never really be in the best interests of an individual patient as compared to the best current standard of care.

We therefore come to another paradox – it is clearly in the interests of all patients, to have more research take place in order to provide the knowledge to underpin their care, but particularly patients with conditions or in situations which are under-researched; but, it is in theory rarely, if ever, in the interests of an individual patient to take part in research as opposed to getting the best standard of individual care. This paradox means that for an individual patient, the best outcome is achieved by refusing to take part in research but making sure everyone else agrees. This is clearly not feasible, nor is the reality of running trials and taking part in them as clear cut. Nevertheless, it is important to realise that the underlying ethical premise of research participation already contains some inherent internal contradictions.

## Living in the real world

There are difficulties with respect to research participation in terms of conflict in ethical principles. The real world is even more complicated, as there are departures in the real world from the assumptions made above. These can broadly be divided into three different categories – constraints due to limitations in access to medical care, constraints due to research design, and constraints due to commercial interests in medical research. Furthermore, the impact of research can be felt not just in terms of the condition and the effects under study. Impact can be found in terms of other aspects of the patient himself, for example his psychological and physical wellbeing; and also his relationships with others and his activities, for example his ability to relate to his family and his ability to learn at school. Therefore any benefit of study may not be limited to the immediate study conditions but has to be analysed in the context of the environment of the patient. The concept of 'benefit' is problematic, as will be discussed later.

In many real life research situations, some treatments undergoing research would be relatively new and may not be publicly funded nor yet licensed for general use. For some patients, for instance those who have failed to respond to conventional treatment or suffer from disorders with no effective treatment, there may be strong inducement to take part in research in order to gain access to the treatment in question. Balanced against this is the argument that research is inherently more beneficial to the individual in these cases, because without the existence of the research study the patients would simply be denied the possibility of being given those treatments in their standard care.

In some cases where medication is already in general use, research participation can nevertheless be extremely beneficial to the individual participants. For example, it has been found that participation in clinical research increases survival for breast cancer patients [22]. This can happen because of the 'real world' of managed healthcare systems, or healthcare systems constrained by limited or uneven distribution of resources. In these non-ideal healthcare systems, some people may not be able to access the best standard of treatment. This occurs in many settings, for example in remote areas in developing countries, and also in areas of poverty and deprivation within industrialised countries. Although such patients would not be getting the best possible individualised care by taking part in research, research protocols tend to guarantee good access to high quality care, support and monitoring for all participants at no financial cost to them.

Pragmatically, it would certainly appear more ethically justifiable to allow research participation if this provides better care than participants would otherwise obtain. However, there could be real concern that this could be a coercive situation if research participation constitutes the best or, worse, the only recourse to treatment for an individual. In such cases there would be undue inducement and little perceived choice concerning research participation, and decisions concerning research participation are unlikely to be altruistic in nature but driven by necessity. Such patients may be left particularly vulnerable, for example, they may not feel able to withdraw from the study because this may compromise their care, or they may feel obligated to the researcher for his or her help.

Health inequalities can lead to differences in ethical standards required for research in different countries and circumstances. In countries or areas where patients may be unable to access high quality healthcare, any research that would provide good medical care for the people might be argued to be highly beneficial and therefore ethically permissible despite the risks involved, whereas the identical research may not be seen as acceptable nor beneficial in well-resourced areas in developed countries. There is then a danger of injustice, that countries and neighbourhoods where there are poorer healthcare facilities may be exploited by researchers from richer countries or institutions [23].

Another aspect of the 'real world' of pharmacological research concerns the design of the studies conducted in these vulnerable populations. To improve both the safety of drugs and the quality of research in paediatrics and in child and adolescent psychiatry, it would be essential to consider special needs of children and adolescents and to investigate the effects of combination therapies (psychopharmacotherapy and psychotherapy, treatment with several medications) [12]. Unfortunately, this is often not reflected in study designs and therefore the quality of treatment in studies may not represent the state of the art [24]. The exclusion criteria of any additional treatment or severe co-morbidity may simultaneously represent both best practice in terms of protection of the vulnerable and severely ill population and a study design which provides a suboptimal treatment strategy [14,25]. Furthermore, by failing to represent the real life circumstances of multiple or concurrent treatments, there are risks that research may fail to provide realistic answers that are helpful to clinicians who need to interpret research results and apply them to their clinical practice.

There are other aspects to living in the real world – the nature of other vested interests, such as commercial interests, in pharmacological research. These will be discussed in a later section.

## Issues of consent and competence

There are two basic ways of considering the ethics of any issue. One is to judge the rightness or morality of any action by the degree of good or harm that results from it, known as utilitarianism; the other is to judge the rightness or morality of any action by how much it conforms with the generally (often tacitly) accepted ethical principles. In general, justifying actions only by outcome alone is not sufficient, as demonstrated by several international conventions regarding human rights which articulate ethical principles concerning rights and duties. With regard to children and adolescents, as legal minors, consent is an area where several different ethical principles can conflict:

• The principle of autonomy suggests that individuals who are competent (that is, able to make their own decisions) should generally give consent for their own treatment. There is an ethical obligation to involve all children in decision-making, according to their maturity. In the legal regulations the obligation of parents to involve their children in decision-making is defined in a way similar to the Convention of Human Rights and Biomedicine of the European Council: the will and the mind of the minor "*shall be taken into consideration as an increasingly determining factor in proportion to his or her age and degree of maturity." *(Article 6, Convention of Human Rights and Biomedicine [26]).

• The principle of best interests, however, suggests that patients, in particular vulnerable patients, should be protected in their best interests, whatever their treatment decisions are. (Article 3: 1, UN Convention of the Rights of the Child [27]: *"In all actions concerning children, whether undertaken by public or private social welfare institutions, courts of law, administrative authorities or legislative bodies, the best interests of the child shall be a primary consideration."*)

• A further principle of protection of children suggests that legal minors are in greater need of protection than other individuals by virtue of their relative immaturity, irrespective of whether they are competent to make their own treatment decisions; therefore they should not be permitted to make major decisions which will seriously harm them (Article 3: 2, UN Convention of the Rights of the Child [27]: *"States Parties undertake to ensure the child such protection and care as is necessary for his or her well-being, taking into account the rights and duties of his or her parents, legal guardians, or other individuals legally responsible for him or her, and, to this end, shall take all appropriate legislative and administrative measures."*)

• There is also a relatively little discussed principle of parental responsibility, which gives parents responsibility for the welfare of their children and therefore a (limited) right to be involved in children's decisions, where it is relevant and necessary to their role as parents, depending on the child's state of development and dependency (see Article 3:2 above and Article 18:1. *"States Parties shall use their best efforts to ensure recognition of the principle that both parents have common responsibilities for the upbringing and development of the child. Parents or, as the case may be, legal guardians, have the primary responsibility for the upbringing and development of the child. The best interests of the child will be their basic concern."*).

• And finally, there is a principle of the importance of family relationships, expressed in the European Convention for Human Rights as 'a right to family life', where both children and parents have a right to family life and the relationships involved (Council of Europe Convention for the Protection of Human Rights and Fundamental Freedoms as amended by Protocol No. 11, Article 8 Right to respect for private and family life [28]: *"Everyone has the right to respect for his private and family life, his home and his correspondence."*)

**Figure 1:**
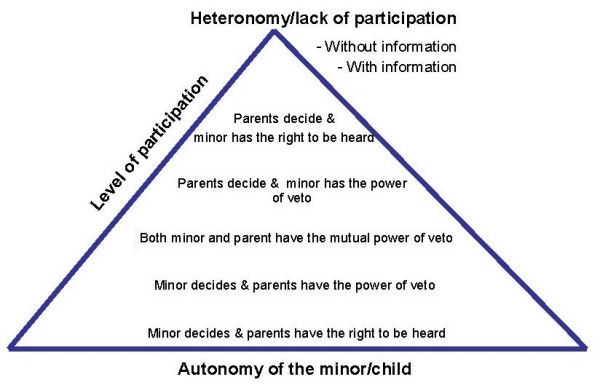
Levels of autonomy and participation of minors in decision making.

Negotiating the issue of consent involves balancing all these different and often opposing principles. Very young children lack the understanding to make their own decisions, so parents usually make decisions for them, and are expected to do so in their best interests. As children develop, their parents and other adults begin to foster their autonomy, so that children should be increasingly able to make, and allowed to make, more decisions for themselves, in proportion to their maturity and the importance of the decisions. Whereas the autonomy of the young child may be less developed, autonomy and the right to autonomous decision-making increases with age as children mature emotionally and intellectually. Children's (and adolescents') rights with regard to decision-making have to be balanced against their ability to deal with the responsibilities that come with it (see Appendix 5). Therefore we can see the autonomy of decision making in minors as a continuum between the extremes of no autonomy and of total autonomy of the minor (see Figure 1) [29]. Parallel to this, their parents have responsibilities with regard to decision-making to an extent that is inversely proportional to whether their children are able to make decisions for themselves. These responsibilities endow the parents with rights to information about their children as appropriate to their involvement.

When children and young people suffer from mental or physical disorders, they become more vulnerable and the issue of protection becomes more important. Because of this vulnerability, the emphasis on protection tends to be greater for research in legal minors, and it is standard for Institutional Research Boards (usually known as IRBs, which are ethics oversight committees) to insist on informed consent from parents as well as assent or consent from the child or young person for participation in research, which is a higher standard for consent than required for standard treatment. This insistence on parental involvement can be ethically problematic for competent young people who may be taking part in research that involves discussing sensitive topics, for example illicit drug use and suicidal intent.

Another area that can be problematic is that of competence. Competence is usually conceptualised as a largely cognitive skill which should be assessed for each specific decision. A person may possess competence to make one sort of decision even if he or she lacks it to make another. There are slightly varying definitions of competence in various legislations as well as various ethical analyses, but in general these include:

• the ability to understand the relevant information;

• the ability to retain the information long enough to arrive at a decision;

• the ability to appreciate its application to oneself;

• the ability to weigh the facts in the balance in order to come to a decision;

• the ability to reason about the treatment options;

• the ability to communicate a choice [30] (see Appendix 6).

Research has shown that children have the competence to make most treatment decisions by the age of 9 years when given simplified information, and by the age of 14 years when given adult-level information [31]. This would suggest that even relatively young children should be able to make their own decisions. However, research has also shown that children and adolescents can have other problems with making autonomous decisions [32]. They are more sensitive to the views of the adults around them and susceptible to pressure as well as worries about offending researchers and parents if they change their minds. Having a mental disorder can have an additional impact upon treatment decision-making for children and adolescents. In some cases, they can become more vulnerable and regress in terms of needing more parental support for decisions they may ordinarily be able to make. Having a mental disorder can have complex effects on autonomy. For example, research suggests that having an eating disorder may distort their sense of identity, values, goals in life and sense of their future [33,34]. Research also suggests that children with attention-deficit/hyperactivity disorder may be influenced by values or hopes of gaining their parents' confidence when deciding whether to participate in a trial which offers them the chance to improve behaviour [Koelch M, Burkert J, Prestel A, Singer H, Schulze U, Fegert JM. The MacArthur Competence Assessment Tool for Clinical Research (MacCAT-CR) in child and adolescent psychiatry. General remarks about the feasibility of the instrument in a special population. Journal of Child and Adolescent Psychopharmacology 2008, submitted].

Even if they have competence to make decisions, because of their close relationships with their parents, many children and adolescents may prefer to have a group or joint decision-making model, making decisions together with parents and other trusted adults. Children and adolescents who are suffering from mental disorders are probably more likely to prefer, and benefit from, a joint decision-making model [35].

## The concept of benefit

Regulations governing interventional research in vulnerable groups such as children and adolescents generally demand that the intervention should benefit the research participant, and pose either or both minimal risk and minimal burden; or, if the risks or burden are greater than minimal, that the participant must obtain a significant benefit at least equivalent to that of standard treatment. Unfortunately, all three concepts of 'benefit', 'minimal risk' and 'minimal burden' are problematic both in terms of the underlying ethical issues as well as definition.

The concept of benefit is deceptively simple. The 'benefit' of a study or of the participation in the study is a measure that resists dichotomisation, so that it can be difficult to be certain whether a person does or does not benefit from research participation. Instead, there is usually a spectrum of magnitudes of potential or actual 'benefit', which can be perceived differently from the perspectives of the researcher, the study outcomes, and the patient. Furthermore, the measurement of benefit can be difficult, as there are three distinct dimensions on which benefit of participation in a study may be measured. These are the probability, the magnitude and the sustainability of benefit [36]. The nature of probability is that even if it is considered likely that participants will benefit from research participation, some individuals may fail to benefit. Magnitude of benefit may vary between participants as well as between studies, and benefit may occur in many different aspects, including some which may be unexpected or difficult to measure. Finally, sustainability of benefit may vary between studies, with some having immediate but short-lived benefit, while others have longer term or even late-onset benefit.

Benefit, therefore, is difficult to define and measure. Benefit has to be assessed both for a study in general terms, and also for the individual study interventions, the study design and investigations or examinations. Benefit has to be further weighed up for each patient in the context of his individual perceptions and values, life situation and stage of disease.

## The concepts of minimal risk and minimal burden

Because of the emphasis on the protection of children and young people, Institutional Research Boards (IRBs) often insist that many protective mechanisms are built into interventional medicinal trials, and research that involves more than a small amount of risk tends to be rejected. In a recent directive, the European Parliament required that 'minimal risk' should be the standard for research trials in children [19]. In this directive, there is a requirement that *"medicinal products for trial may be administered to all such individuals only when there are grounds for assuming that the direct benefit to the patient outweighs the risks"*. A prerequisite for research is that *"some direct benefit for the group of patients is obtained from the clinical trial and only where such research is essential to validate data obtained in clinical trials on persons able to give informed consent or by other research methods; additionally, such research should either relate directly to a clinical condition from which the minor concerned suffers or be of such a nature that it can only be carried out on minors"*. As the directive must be implemented by members of the European Union in national law, each member state will have to adapt these sentences into its own law.

The definition of this notion of 'minimal risk' is problematic. There are variations in the definition across countries:

• In the United States (The Common Rule, 1991):

- " [risks] ordinarily encountered in daily life or during the performance of routine physical or psychological examinations or tests"

• In Canada (2005):

- "no greater than those encountered by the subject in those aspects of his or her everyday life that relate to the research"

• In Europe – the Council of Europe (2005) (minimal risk and minimal burden):

- "*very slight and temporary negative impact on the [person's] health" *and *"discomfort [i.e., burden] will be temporary and very slight"*

• In the United Kingdom (2004):

- "procedures such as questioning, observing, and measuring children [and] obtaining bodily fluids without invasive intervention; [no] more than a very slight and temporary negative impact on [child's] health"

- Low risk: *"might cause no more than brief pain or tenderness, small bruises or scars, or very slight, temporary distress; e.g., a blood test"*

• In Germany (German Drug Code: 12. Amendment German Drug Code §41 (2), 2004):

- "If it is expected that the intervention at most leads to a very slight and temporary impairment of the health of the subject. Minimal burden is seen if the intervention causes discomfort which is at the most temporary and very slight."

There is some conceptual confusion around the idea of 'minimal risk'. Is 'minimal risk' supposed to mean 'minimal distress and suffering'? This interpretation has some merit, as we would wish to inflict minimal pain and distress, irrespective of benefit or risk, particularly for non-consenting individuals such as babies and young children who would have little or no appreciation of the research rationale or benefits. Or is 'minimal risk' instead supposed to mean 'not much greater risk of harm as compared to ordinary life and ordinary treatment'? This alternative interpretation also has merit, particularly when looking at the issue of preserving the best interests of the vulnerable participant. The two definitions would overlap in real life, but are conceptually distinct. For example, a procedure producing a great deal of benefit and relatively little medium to long term harm may be very distressing to an individual. Yet, another procedure exposing an individual to much greater risk of harm than he or she would ordinarily experience may evoke little distress or suffering. Also, the standard treatment may cause considerable distress and side effects, and the alternative research treatment may have greater risks in terms of having less benefit and more toxic effects, but be a great deal more pleasant than the currently available treatment.

What are study procedures which can be considered as minimal burden for the patient or have minimal risk for him? Examples that have been given in the regulations for minimal risk and burden are measuring, weighing, and the drawing additional a minimal quantum of blood by an already existing venous access. However, even single and small additional blood drawing may be already a risk which is increased over minimal risk, if the standard treatment has already required a large number of blood drawings or a large volume. Also a repetitive examination which would count in case of a single procedure as a minimal burden may be more than a minimal burden if it is conducted with a high frequency.

The variation between countries about whether they interpret 'minimal risk' to mean lower distress, lower risk, or both, means that there will be confusion about what risk is considered acceptable, and little consistency of the ethical standards adopted by research studies across these countries.

The minimal risk has to be examined in the individual case of each study and indeed each patient. There is no final agreement about the terms and therefore a discussion in study teams and Institutional Research Boards (IRBs) may lead to a different consensus for each individual study. This may disconcert researchers who cannot refer to a standard, but this reflects the general difficulties of applying ethics in research, that general rules are often difficult if not impossible to provide. The researcher himself has the obligation to consider the ethical aspects of the study he plans, and to be prepared to justify his approach. The differences in national guidelines and laws in their definition of minimal risk and burden also requires an individual discussion of study procedures within each jurisdiction to ensure conformity with national law and its interpretation [37,38].

## Ethical issues in research design

There are two different but related ways in which research design can be ethically problematic:

1. when the design itself may raise ethical concerns, for example when participants may be at risk or even harmed (see discussion above of benefit and risk); and

2. when the design is scientifically sub-optimal, which makes it ethically dubious because we should not subject research participants, particularly those from vulnerable groups, to research which may not be adding good quality evidence to the scientific body of knowledge.

The best design for testing the effect of pharmacological interventions is the randomised controlled trial of a drug against a placebo control (E.g., [12,39,40]), Unfortunately, except in the increasingly rare situations where there are no drugs which are in general use or have any known efficacy for the disorder, there are serious ethical issues involved in subjecting participants to a placebo control because this effectively means denying them active treatment for the duration of the trial. There have been some research designs which attempt to compensate for this, for example double cross-over trials. However, even this can only be done in a limited number of disorders, for example those where a period without treatment is considered safe.

**Figure 2:**
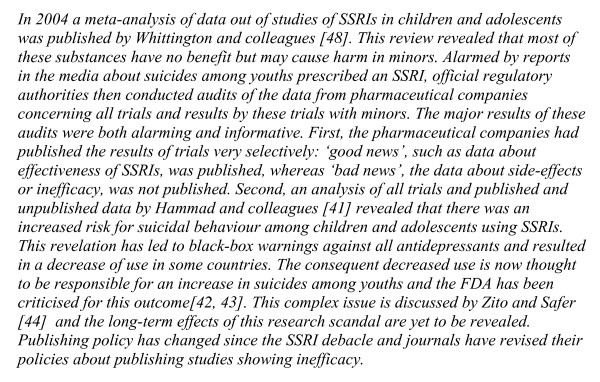
Text box: 'The sad story of selective serotonin reuptake inhibitors (SSRIs) in children and adolescents'.

Limitations in research design can lead to serious difficulties. In adolescent psychiatry, most of the drug trials involving Selective Serotonin Reuptake Inhibitor (SSRIs) pitted one SSRI against another, but almost all have been discredited because of failures to report adverse events such as suicide (see Figure 2) [41-44]. This has left the evidence base very shaky and the withdrawal of all except one SSRI, Fluoxetine, for the treatment of depression in adolescents.

Another way in which research design may be scientifically suboptimal is when the comparisons being made do not reflect clinical reality. This is highly relevant to child and adolescent mental health, where pharmacological treatments are rarely used in isolation, and are usually combined with a psychological therapy. There are, however, commercial constraints which explain why naturalistic combined treatment trials are rarely funded and therefore rarely conducted. This will be discussed in the next section.

## Ethical issues in commercial research interests

Pharmacological research trials are extremely expensive to set up and run. The possibility of legislation to increase the availability of approved and safe drugs for children has been discussed in the USA and Europe [11,14-17,45-47]. An essential difference exists between the US and Europe in funding clinical trials with minors. In Europe, given the general paucity of public research funds and the expense of interventional trials, publicly funded pharmacological research is still relatively rare. Commercial pharmaceutical companies, however, have strong vested interests in finding their products, particularly new products still under patent, to be superior to other medications. The ultimate bottom line for pharmaceutical companies, which are commercial institutions with accountability of management to shareholders, is to create profit. Coupled with the common problem of publication bias because trials with negative results are less interesting and therefore harder to publish than positive ones, this can create significant skewing of results (see for example the problems accompanied with SSRI in children) [48-50]. Skewed results showing a spurious effect can alter clinical practice and potentially harm patients as ineffective medication may be prescribed which would expose patients to risks of medication without the benefits.

Researchers who are employees of pharmaceutical companies can experience moral dilemmas between maintaining scientific objectivity and the obligation to try to produce positive results that can help their employers achieve targets. Researchers who have their research or teaching activities funded by pharmaceutical companies may also experience this moral dilemma. There is a growing movement against commercially funded research and researchers, with independent experts, researchers and research being favoured as less biased [51,52]. In the past, pharmaceutical companies have been lavish in funding teaching, seminars and academic activities such as conference attendance for physicians. There is, however, evidence that these commercial sponsorships have an impact on prescribing practices of physicians [49,52-55]. There has a growing suspicion of commercial interests as unethical and a backlash amongst doctors against pharmaceutical sponsorship and commercially funded research.

A further ethical dilemma in commercially driven research is that where there is relatively low financial gain to be had from conducting research, pharmaceutical companies are less likely to fund it. This is the case for psychopharmacological research in children and adolescents. Once a new drug is licensed for use in adults, given the lack of research evidence for drugs in adolescents and particularly children, child and adolescent psychiatrists will begin to prescribe these drugs 'off-label' to their patients. To attempt to conduct research in children and adolescents to obtain licences for these age groups would be both more complex and therefore expensive for companies, and may also provide minimal additional commercial yield.

Yet another ethically relevant point is the need for combined treatment strategies in child and adolescent psychiatry. To improve the safety of drugs in paediatric and adolescent psychiatry, it will be essential to consider special needs of children and adolescents and to investigate the effects of combination therapies (psychopharmacotherapy and psychotherapy, or treatment with several medications). The really essential evidence of the last decade has been found in publicly funded trials on attention-deficit/hyperactivity disorder (ADHD) and in depression (major depressive disorder, or MDD). The NIMH Multimodal Treatment Study of ADHD (also known as the MTA study) and the NIMH-funded Treatment for Adolescents with Depression Study (TADS) compared both psychotherapy and psychopharmacotherapy with the combined treatment, which is a more naturalistic approach than having two solely pharmacotherapy treatment arms [56,57].

Pharmaceutical companies have not tended to be interested in funding multimodal interventional research as their primary interest lies in the superiority of one pharmaceutical product over another. Furthermore, in the case of comparing two substances from two different manufacturers, patent aspects may be an important hindrance. There has therefore been little research funding from the companies for this particularly vulnerable patient group. The new European legislation has the potential to help facilitate such naturalistic trials, but to what extent such desiderata are taken into consideration by the EU regulation is being critically discussed [11,58].

As much as commercial interests may affect research, it would be even more unethical, in addition to being unfeasible, to discount or seek to dismiss all commercially funded research. It is unrealistic to expect that pharmaceutical research will be totally or even mainly funded by non-commercial organisations. Nor is it realistic to expect an altruistic attitude to research funding from commercial companies. Nevertheless, it may be possible to persuade commercial companies that more naturalistic multimodal or combination therapy trials are both commercially viable and advantageous. Both the TADS and the ADAPT naturalistic trials have been more successful in providing strong evidence for efficacy of drugs than previous trials [24,59]. Convincing evidence of efficacy in naturalistic settings is more likely to lead to greater acceptance in prescribing the relevant medication. For a way forward to solve these problems a discourse between ethicists, child and adolescent psychiatrists and industry could create ideas of ethically sound and scientifically justified study programs and protocols, which fulfil naturalistic treatment conditions.

## Finding a way forward

In the light of so many difficult, intertwined and disparate ethical dilemmas, it is tempting to despair of whether any psychopharmacological research in children and adolescents can ever be ethically sound. It is important that the exploration of ethical dilemmas should not end with the naming of a long list of problems and a metaphorical wringing of hands, but that some thought and energy should be given to possible ways forward.

The positive way forward in the development of ethically sound psychopharmacological research in children and adolescents is to bring research ethics into the heart of research development and design itself, rather than just using research ethics as an oversight mechanism to identify flaws and potentially block research, as is commonly the case or perception of researchers. Ethicists themselves can have a range of attitudes between 'conservative' views which may tend to protect patients but at the risk of stifling research, to 'liberal' views which may tend to promote research but at the risk of accepting exposure of patients to more distress or risk. As previously discussed, past abuse has led to a conservative attitude to research. It may be time to acknowledge this legacy and to shift to a more balanced attitude which tries to find a middle ground between overly conservative and overly liberal ethical views towards research, and to foster a culture where both excessive conservatism and liberalism can be openly challenged and discussed.

In order for ethics to become a helpful and integral part of research, it is important that there should be a gradual change in culture and approach involving hearts and minds, rather than a change in regulations that increases the burden on researchers. Regulation by its nature is about ensuring a minimal acceptable standard; in contrast, bringing ethics into the heart of research paradigms should be about creating a maximal, gold standard of ethical and ethically-conducted research.

There are three different and complementary ways in which this can be achieved:

• A comprehensive ethical analysis which takes into account the different and conflicting ethical principles relevant to psychopharmacological research in children and adolescents;

• Conducting empirical ethics research into issues concerning psychopharmacological research in children and adolescents; and

• Building in ethical thinking and analysis into psychopharmacological research in children and adolescents.

### i. A comprehensive ethical analysis

Most of the ethical principles which are relevant to psychopharmacological research in children and adolescents have been developed in other fields and for other situations, which is why there has been relatively little resolution of their conflicting implications. For example, the principle of autonomy was developed for autonomous adult patients based on an individual autonomy model which is in turn based on the idea of individual patients making rational choices. The principle of protection of children, in contrast, has been developed from a tradition of protecting children from abuse and exploitation, in a tradition where children have been seen as having little voice or control over their situations with respect to adults who are disposed to harm rather than protect them.

A comprehensive ethical analysis would involve naming and exploring all the different principles that are relevant to this field. It would also involve having debates involving ethicists, researchers, clinicians, policymakers and patients to decide whether certain principles are imperative and immutable, and if so, which ones; or whether all the principles are relative and variable. By acknowledging the difficulties of navigating so many different and often conflicting principles, it can become possible to explicitly compare and weigh them against each other, and to be able to accommodate different situations which may require different resolutions. For example, should upholding the notion of 'minimal risk' always be paramount? Or are there situations, such as in research with competent older adolescents and disorders without any known treatment, where taking greater risks may possibly be balanced by having greater potential benefits, or where protection may take second place to fully informed consent and great potential scientific as well as individual benefit? There needs to be open dialogue and debate about these tensions, between researchers, ethicists, research funders and policy-makers.

A final merit of a comprehensive ethical analysis is that other ethical considerations which have not been prominent in the ethical debates concerning psychopharmacological research in children and adolescents can be raised. One example of this is the issue of Altruism. The current system of ethics oversight of research, in its preoccupation with protection of research participants, does not allow for much consideration of altruism on the part of the participant. This is particularly the case when the research participants are considered vulnerable or may lack competence. When this happens, the only rationale which appears to be an acceptable justification for research is benefit for the individual or, at most, for others who suffer from the same condition. People, however, often have more noble motives. Many children and young people are highly idealistic and altruistic, and even though their protection is important, it is also important to allow children and young people opportunities to develop a sense of citizenship and make contributions to society. Research shows that children and their parents are prepared to consider undergoing some risk or discomfort in order to participate in medical research which would be of benefit to others but not themselves [60].

### ii. Empirical ethics research

Empirical ethics research is a relatively novel field, where ethicists use empirical research methods to observe the 'real world' dilemmas that occur around particular issues, and develop ethical analyses based on these research findings. Empirical ethics has the merit of being grounded in real life ethical dilemmas, as opposed to theoretical reasoning of what is morally right or wrong. Theoretical reasoning, while very valuable, tends to be dogmatic and may be experienced as unhelpful or out of touch by those struggling with complex ethical dilemmas as well as constraints such as limited resources. Empirical ethics research, in contrast, is more likely to capture the full complexity of their dilemmas and the concurrent constraints, and is therefore more likely to be able to generate helpful analyses and pragmatic suggestions for ethical resolution [61].

Examples of empirical ethics research that would be useful to do are:

• Competence to consent to research in children and adolescents who suffer from mental disorders;

• The views of children, young people and their parents about research participation and what the ethical issues may be for them;

• The understanding of the nature of altruism and its place in psychopharmacological research in children and adolescents;

• The experiences of children, young people and their parents in participating in psychopharmacological research;

• The ethical dilemmas that research staff and clinical teams experience in the course of such research.

Such empirical research can be done alongside psychopharmacological research in children and adolescents, as 'bolt-on' modules of research which can occur alongside the core research.

### iii. Building in ethical thinking and analysis into psychopharmacological research in children and adolescents

Researchers, particularly those who labour under strict ethical oversight systems, may be driven to consider research ethics as onerous and designed to stifle research. A more healthy approach is to embrace research ethics and to consciously design ethical input into research methodology. This can be done in several ways:

• Having a research ethicist involved in developing the research design can help to ensure the development of ethical research designs and thoughtful consideration of the various ethical tensions.

• Having a steering group involving patients and parents can help researchers identify, from their perspective, what might be the relevant ethical issues, and, equally, what might be non-issues. The steering group can also help the researchers to design the study in such a way as to minimize ethical problems.

• Having a research ethicist as a consultant during the conduct of the study can ensure that any issues that arise can be considered properly and effectively dealt with.

• Having an ethicist on the data management committee which has oversight of the emerging data; data management committees may have to consider issues such as the welfare of patients in the arm of a study which is showing less benefit.

• Conducting preliminary empirical ethics research, such as investigating patients' and parents' views of a controversial research design or a potentially risky but beneficial treatment, may be helpful in determining whether such research could or should be undertaken, and how this can best be achieved. Pragmatically, such preliminary research can also assist in demonstrating to ethics oversight committees that the ethical ramifications have been taken seriously and adequately explored.

• Bolting on empirical research studies to the main study, as described above, which has a protective effect by ensuring that the ethical dilemmas that emerge are being studied, in addition to adding to the body of knowledge about the ethics of research.

## Conclusion

Research in psychopharmacology for children and adolescents is fraught with ethical problems and tensions. What is needed is both an increased awareness of the ethical issues and tensions which permeate such research, as well as a willingness to acknowledge these issues and to invest time and commitment to exploring them. Just as we need more psychopharmacological research in children and adolescents in order to develop a sound understanding of how to best treat children and adolescents with mental disorders, so we need more ethical analysis and ethics research in order to develop a sound understand of what constitutes good, ethical psychopharmacological research in children and adolescents.

## Competing interests

JT has received research grants from the Wellcome Trust. MK has received research grants from the Eli Lilly International Foundation; the German Federal Ministry of Families, Women Seniors and Youth; the Federal Justice Department of Switzerland; and Boehringer Ingelheim, European Academy. He was an investigator in clinical trials funded by Astra Zeneca, Janssen-Cilag and Eli Lilly. He was also a recipient of travel grants provided by Janssen-Cilag, the University of Rostock, DGKJPP, UCB and some welfare institutions.

## Authors' contributions

Both authors have contributed equally to the manuscript and are entirely responsible for the scientific content of it. JOAT created the first draft. Both authors read and approved the final manuscript.

## Appendix 1

Introduction 2. *"It is the duty of the physician to promote and safeguard the health of the people. The physician's knowledge and conscience are dedicated to the fulfilment of this duty" *[1].

## Appendix 2

2) Introduction 4. *"Medical progress is based on research which ultimately must rest in part on experimentation involving human subjects" *[1].

## Appendix 3

3) Sentence 3 of the preface: *"Persons who are incapable of giving legal consent to clinical trials should be given special protection. It is incumbent on the Member States to lay down rules to this effect. Such persons may not be included in clinical trials if the same results can be obtained using persons capable of giving consent" *[19].

## Appendix 4

4) Sentence 3 of the preface of the text of the EU Directive 2001/20: *"Normally these persons should be included in clinical trials only when there are grounds for expecting that the administering of the medicinal product would be of direct benefit to the patient, thereby outweighing the risks." *Article 4 of the Directive: *"(g) clinical trials have been designed to minimise pain, discomfort, fear and any other foreseeable risk in relation to the disease and developmental stage; both the risk threshold and the degree of distress have to be specially defined and constantly monitored." *Further aspects are found in ICH E6 §4.8.14 and the U.S. Code of Federal Regulations: Title 45: Public Welfare: 45 C.F.R. §46.405, Research involving greater than minimal risk but presenting the prospect of direct benefit to the individual subjects [19-21].

## Appendix 5

5) In Re:W (A Minor)(Medical Treatment: Court's Jurisdiction) [1992] WLR 758–782: *"Adolescence is a period of progressive transition from childhood to adulthood and as experience in life is acquired and intelligence and understanding grow, so will the scope of decision-making which should be left to the minor, for it is only in making the decisions and experiencing the consequences that decision-making skills will be acquired. As I put it in the course of the argument and as I sincerely believe, "good parenting involves giving adolescents as much rope as they can handle without an unacceptable risk that they will hang themselves"." *(Page 770, extract from the judgement of Lord Donaldson of Lymington, in an English family court case concerning parental consent and courts overriding the treatment refusal of a 16 year old girl with anorexia nervosa).

## Appendix 6

6) Two examples of differing definitions of decision-making in/competence in law are: *"incapable of (a) acting; or (b) making decisions; or (c) communicating decisions; or (d) understanding decisions; or (e) retaining the memory of decisions." *(Adults with Incapacity (Scotland) Act 2000); *"a person is unable to make a decision for himself if he is unable (a) to understand the information relevant to the decision, (b) to retain that information, (c) to use or weigh that information as part of the process of making the decision, or (d) to communicate his decision (whether by talking, using sign language or any other means)." *(Mental Capacity Act 2005, United Kingdom)
